# Concurrent Occurrence of Tumor in Colon and Small Bowel following Intestinal Obstruction: A Case Report and Review of the Literature

**DOI:** 10.1155/2016/8591697

**Published:** 2016-04-20

**Authors:** Seyed Mohammad Reza Nejatollahi, Omid Etemad

**Affiliations:** ^1^The Division of Hepatobiliary & Organ Transplantation, Taleghani Hospital, Shahid Beheshti University of Medical Sciences, Tehran, Iran; ^2^Shahid Beheshti University of Medical Sciences, Tehran, Iran

## Abstract

An intestinal obstruction occurs when either the small or large intestine is partly or completely blocked so it prevents passing the food or fluid through the small/large bowel. This blockage is due to the existence of a mechanical obstruction such as foreign material, mass, hernia, or volvulus. Common symptoms include cramping pain, nausea and vomiting, changes in bowel habits, inability to pass stool, and lack of gas. We present a case of an 83-year-old man who had been referred to Taleghani Hospital with symptoms of bowel obstruction. He underwent the surgery. The findings of exploration of the entire abdomen showed two types of mass separately in two different organs. In postoperative workup, pathology reported two types of tumors (adenocarcinoma and neuroendocrine tumors).

## 1. Introduction

An Intestinal obstruction occurs when either the small or large intestine is partly or completely blocked so it prevents passing the food or fluid through the small/large bowel. This condition would cause inability to pass food, fluid, and gas in the digestive tract. The obstacle could be a foreign material or the result of volvulus, hernia, or tumor growth [1].

Bowel obstruction could also be a result of abdominal infection or inflammation caused by conditions such as diverticulitis. In patients with Crohn's disease or peritoneal carcinomatosis, the risk of bowel obstruction may arise [2].

Some symptoms of this disease include abdominal cramping, abdominal distension, diarrhea, or inability to have bowel habits [3]. Cancer is suspected in patients older than 60 years who have experienced changes in their bowel habits for more than six weeks [4], with nausea, vomiting, and inability to pass gas or stool [5]. In such cases, first the mechanical factors and then nonmechanical factors must be examined. For this aim, physical examinations, X-ray, CT scan, and lab test are used.

Intravenous hydration and correcting electrolyte levels are the first treatment for such patients and then, according to the nature of bowel obstruction, the curative treatment will be begun [1].

## 2. Case Report

**Figure 1:**
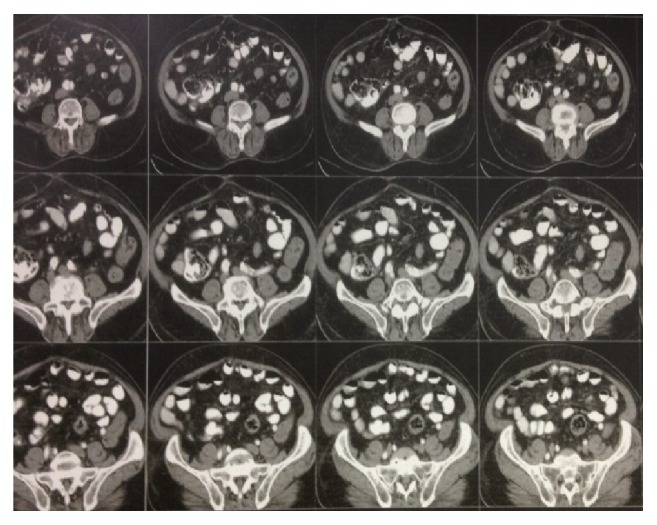
CT scan 5 months before surgery.

This is reportage of an 83-year-old man who had experienced changes in bowel habits with feeling of diffuse abdominal pain aggravated by eating and relieved by passing stool from 5 months before. Due to these symptoms, colonoscopy was planned for him, but because of the lack of consent, CT scan of the abdomen/pelvis was performed. As you can see in Figure 1, the CT scan reported significant thickening in the sigmoid colon.

**Figure 2:**
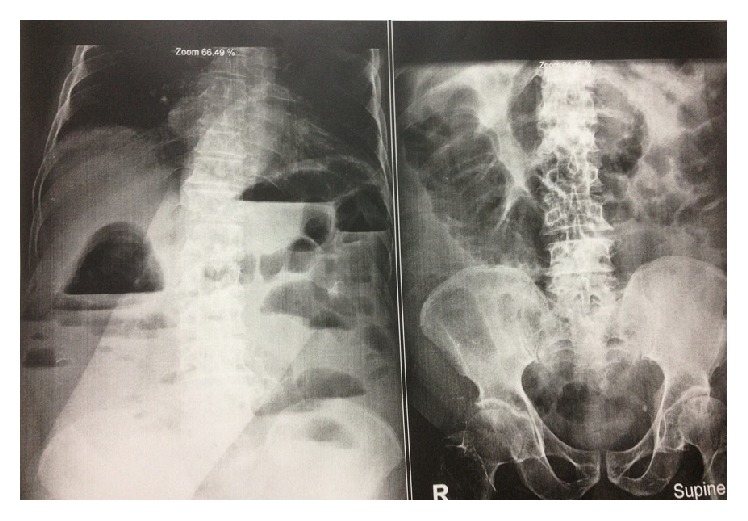
Abdominal X-ray (supine and upright) just before surgery.

Due to increasing abdominal pain, nausea, vomiting, and inability to pass gas and stool for six days, the patient was referred to Taleghani Hospital of Tehran. In Figure 2, the abdominal X-ray showed multiple air-fluid levels. After precise examination and diagnosis of bowel obstruction, the patient underwent surgery.

Intraoperative findings indicated bowel obstruction due to a mass that was located at a distance of 20 cm from the rectosigmoid junction in the sigmoid region, dilation of the colon and small intestine, and another mass with dimensions of 2 × 2 cm that was located at a distance of 80 cm from the ileocecal valve in the ileum region.

**Figure 3:**
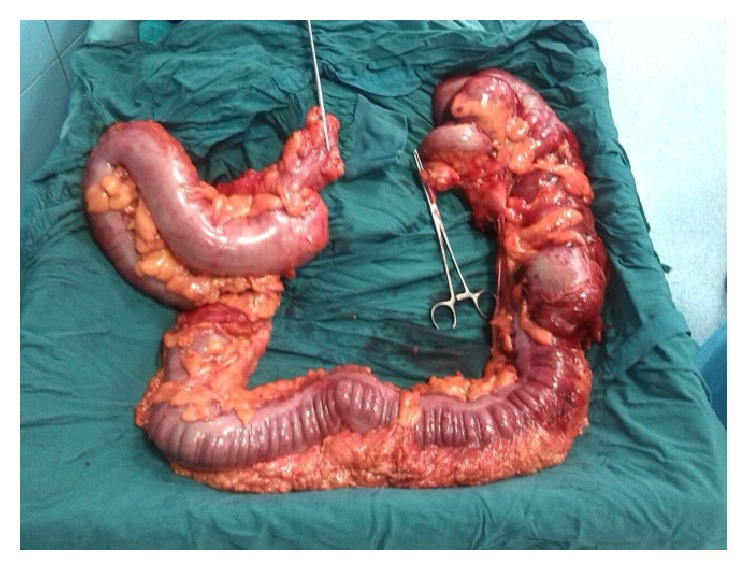
Total colectomy.

The patient underwent the small bowel segmental resection and primary anastomosis and total abdominal colectomy because of the severe dilatation of the proximal part of the colon and ischemic changes of the cecum with ileorectal anastomosis (end-to-end) was done for him. In postoperative workup, there was no sign of metastasis and he was discharged from the hospital after six days with good general conditions.  Figure 3 shows the resected colon after total colectomy.

## 3. Pathologic Findings

The pathologic findings showed existence of two tumors as the following.

(1) The small bowel wall was infiltrated by a neoplasm composed of monotonous small round cells with small nucleoli, salt and pepper chromatin, and moderated finely granular cytoplasm arranged in nesting and trabecular structure.

Well-differentiated neuroendocrine tumor with lymphovascular invasion and size of 2 cm with greatest dimension and mitotic rate of less than 1/10 hpf was seen in the ileum site. Tumor invades the subserosa tissue without involvement of the visceral peritoneum (pT3).

(2) There was a well-differentiated adenocarcinoma with lymphovascular invasion and size of 5.5 cm in the greatest dimension in the sigmoid-colon region with microsatellite instability and mucinous production (20% of tumor). Tumor invades the subserosal tissue and 21 lymph nodes were removed (T3 N0).

Due to the existence of an obstructive tumor and lymphovascular invasion, the patient was a candidate for chemotherapy. But because of some factors such as age and his disability to tolerate the conditions of chemotherapy, chemotherapy was not done for him.

## 4. Discussion

More than 575.000 people die of cancer and more than 1.5 million people are diagnosed with cancer every year in the USA. According to the World Health Organization (WHO), the numbers of new cancer cases are expected to be raised by about 70% over the next 20 years [6]. Surgery is a preferred treatment for many types of cancer.

According to studies in the USA, each year an estimated 8.000 people in the USA are diagnosed with a neuroendocrine tumor that starts in the gastrointestinal tract which includes the stomach, intestine, appendix, colon, or rectum [7].

Well-differentiated neuroendocrine tumors have cells that do not look very abnormal and are not multiplying rapidly and sometimes the only way to know that a mass is a neuroendocrine cancer is when it spreads to the other organs or tissues [8].

In some cases, there is a possibility of existence of some other benign tumor in the other organs. Accordingly the surgeon must check the possibility of other masses in the abdomen [9]. Studies show neuroendocrine tumors in the rectum and colon are rare [10]. Studies have estimated the annual incidence of clinically significant neuroendocrine tumors is approximately 2.5–5 per 100,000 [11]. More prevalence of neuroendocrine tumors is in the small bowel and often in the ileum (about 70%) [12].

The synchronous occurrence of two differentiated tumors in a single patient is rare. So far, there are several reported cases that had two differentiated tumors in two or several other organs simultaneously [8, 13–15].

Ferrando Marco and his colleagues reported the case of a 64-year-old man with periampullary collision tumor, in which a duodenal-wall carcinoid and an adenocarcinoma of the head of the pancreas coexisted [9].

Akiba and his colleagues reported a case of a 64-year-old man who was found to have EEBV-associated gastric carcinoma with primary gastric extranodal marginal zone lymphoma of mucosa-associated lymphoid tissue [16].

Kleist and his colleagues reported two collision tumors containing a gastrointestinal stromal tumor with intermingling elements of gastric adenocarcinoma [17].

Jang and his colleagues reported a case of a 70-year-old man found to have two separated masses which were observed in the proximal ascending colon containing two separated well-differentiated neuroendocrine tumors with necrosis and increased mitosis [18].

Singh and his colleagues reported a case of a 52-year-old found to have a mass at the base of the appendix. On microscopic examination of the tumor, mixed adenocarcinoma and carcinoid were identified [19].

Van Kerkhóve and his colleagues reported a case of a 72-year-old female found to have a rare ileal collision tumor consisting of an adenocarcinoma and a small cell neuroendocrine tumor with peritoneal metastasis of neuroendocrine origin and coincidental benign lesions on both ovaries [20].

According to the studies, 15–35% of patients with neuroendocrine cancer in the ileum had more than one tumor and 50–60% of them survived about 5 years after surgery (85% if the tumor was confined to the bowel wall and 5% in case of serosal invasion) [21].

In 15–30% of cases with ileum neuroendocrine tumor, there is more than one tumor, and 15–29% of tumors are associated with other noncarcinoid malignancies [22].

## 5. Conclusion

Neuroendocrine tumors are neoplasms that are raised from cells of endocrine and nervous system. They could be malignant or benign and most commonly occur in the intestine and will spread to other organs. Ileal neuroendocrine tumors often grow in the distal part of the ileum. In 15–30% of cases, there is more than one tumor, and 15–29% of tumors are associated with the other noncarcinoid malignancies.

Diagnosis must be done by using laboratory tests, X-ray, CT scan, and endoscopy. Surgery is a definitive treatment in the early stages. Patient's age, the exact location, dimension and differentiation of tumor, and lymphovascular invasion are parameters which affect the progression of the disease.

In this case report, the patient with diagnosis of the bowel obstruction underwent surgery and we found a mass in the sigmoid region with pathologic diagnosis of adenocarcinoma and another mass in the ileum region with pathologic diagnosis of a neuroendocrine tumor.

Due to the increased prevalence of malignant tumors, checking the entire of the digestive system according to existing protocols in similar cases and not forgetting to consider the possibility of coexisting malignant tumors to find and treat them at an early stage are recommended.
